# Assessment of potentially inappropriate medication use among geriatric outpatients in the Slovak Republic

**DOI:** 10.1186/s12877-023-04260-y

**Published:** 2023-09-15

**Authors:** Stanislava Kosirova, Jana Urbankova, Jan Klimas, Tatiana Foltanova

**Affiliations:** Faculty of Pharmacy, Department of Pharmacology and Toxicology, Comenius University Bratislava, Odbojarov 10, Bratislava, 83104 Slovak Republic

**Keywords:** Potentially inappropriate medication, Geriatric patients, EU(7) PIM list

## Abstract

**Background:**

Potentially inappropriate medication (PIM) use is a highly prevalent problem among older people, making it challenging to improve patient safety. The aim of this study was to assess the use of PIMs among geriatric outpatients (OUTs) in the Slovak Republic according to the EU(7) PIM list and to identify the differences in PIM prescriptions among general practitioners (GPs), internists (INTs) and geriatricians (GERs).

**Methods:**

In total, 449 patients (65 years and older) from 4 medical centres who were in the care of GPs (32.5%), INTs (22.7%) or GERs (44.8%) were included in this retrospective analysis. Data were collected from 1.12.2019–31.3.2020. PIMs were identified according to the EU(7) PIM list from patients’ records. PIM prescriptions by GPs, INTs and GERs were assessed. All obtained data were statistically analysed.

**Results:**

Polypharmacy (68.8% of patients), and PIM use (73% of patients) were observed. The mean number of all prescribed drugs was 6.7 ± 0.2 drugs per day/patient. The mean number of prescribed PIMs was 1.7 ± 0.1 PIMs per day/patient. Drugs from Anatomical Therapeutic Chemical (ATC) classes C, N and A accounted for the greatest number of PIMs. Significantly higher numbers of prescribed drugs as well as PIMs were prescribed by GPs than INTs or GERs. There were 4.2 times higher odds of being prescribed PIMs by GPs than by GERs (p < 0.001).

**Conclusions:**

Polypharmacy and overprescription of PIMs were identified among geriatric patients in our study. We found a positive relationship between the number of prescribed drugs and PIMs. The lowest odds of being prescribed PIMs were observed among those who were in the care of a geriatrician. The absence of geriatricians and lack of information about PIMs among general practitioners leads to high rates of polypharmacy and overuse of potentially inappropriate medications in geriatric patients in the Slovak Republic.

**Supplementary Information:**

The online version contains supplementary material available at 10.1186/s12877-023-04260-y.

## Background

The increased life expectancy of the population has resulted in a growing proportion of people over 60 years of age. According to the WHO, between 2015 and 2050, the proportion of the world’s population over 60 years will nearly double from 12 to 22% [1]. This brings new challenges for health care systems, the environment, and society. Due to the physiological changes of the human body with aging, as well as due to multimorbidity, proper pharmacotherapy for elderly individuals is a complex and serious task. Multimorbidity leads to an increase in the number of prescribed drugs and ultimately to polypharmacy [2]. Polypharmacy is associated with increased prescriptions of potentially inappropriate medications (PIMs) and thus an increased risk of adverse drug reactions and other drug-related problems [3–5]. PIM use is often associated with negative health outcomes, including hospitalization and mortality [6], functional decline and falls [7].

Medications that are listed as potentially inappropriate for geriatric patients have insufficient evidence of efficacy, cause a higher risk of adverse effects in patients, and their risk to the patient outweighs the benefit [8]. To increase the safety of pharmacotherapy for elderly individuals, appropriate prescribing is highly recommended. It is important not to simply reduce the number of prescribed drugs [9] but to deprescribe inappropriate medications and prevent prescribing cascades [10] as well [11]. In this effort, lists of potentially inappropriate medications are useful. There are tools or criteria for assessing inappropriate prescribing based on national criteria and the availability of drugs at the national level [12]. The criteria can be explicit, implicit, or mixed. Explicit criteria represent lists of PIMs and could be used without a clinical judgement of the patient [13]. These criteria could be simply applied for the evaluation of appropriate drug prescriptions for patients. Implicit criteria are patient specific, and for their application, clinical judgement is needed [14].

The first explicit PIM list was published in 1991 in the USA, and it was the Beers Criteria list [15]. Since its publication, the American Geriatrics Society has reviewed and published the Beers Criteria list every three years, and the latest update was in 2023 [16]. Although there are more than 200 medications or medication classes on the Beers list, half of them are not used in Europe. This has led to the development of other tools, such as the Laroche criteria [17], PRISCUS list [18], FORTA [19], STOPP/START criteria [20] and the European list of PIMs (EU(7) PIM list) [21], which are more suitable for use in European countries. The EU(7) PIM list consists of 282 substances or drug classes from 34 therapeutic groups that are PIMs for older people; some of them are restricted to a certain dose or duration of use. The EU(7) PIM list contains a description of dose adjustments and therapeutic alternatives that have been prioritized by experts [21].

Many European countries, e.g. France, Finland, Switzerland, Sweden, Portugal, Bulgaria and the Czech Republic, have assessed the use of PIMs in elderly individuals [5, 22–27]. The Slovak Republic thus remains one of the few European countries from which data about PIM use in elderly individuals are limited [27]. Our previous pilot study in nursing homes in the Slovak Republic showed high rates of polypharmacy (83%) and an alarmingly high rate of PIM use (91%) [28]. However, the rate of polypharmacy and the use of PIMs in ambulatory settings are still unknown. Therefore, we decided to fill this gap with information about PIM use among ambulatory outpatients. This study aimed to assess the use of potentially inappropriate medications among geriatric outpatients (OUTs) in the Slovak Republic according to the EU(7) PIM list and identify the differences between the prescription of PIMs by general practitioners, internists and geriatricians. We believe that more detailed information will be of great importance when targeted interventions are prepared and implemented by health authorities and policy-makers.

## Methods

### Study design and setting

**Fig. 1:**
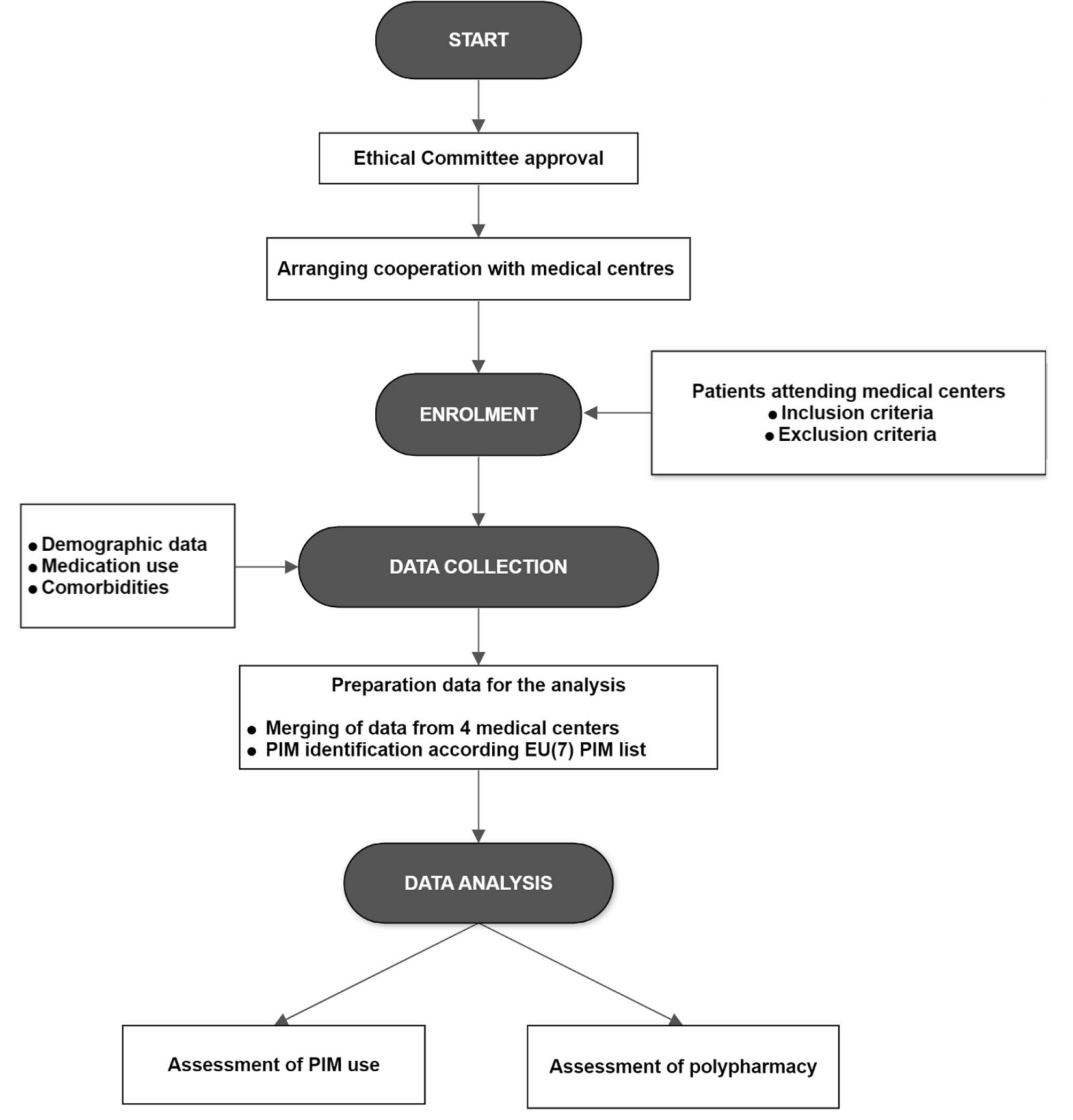
Flowchart of the study. *PIM - potentially inappropriate medication*

This was a retrospective population-based study among geriatric outpatients in 4 medical centres in the Slovak Republic (Kosice, Puchov, Stara Lubovna, Vranov nad Toplou) (Fig. 1). Patients were attending the medical centres from 1.11.2019–31.12.2019. The data were collected by four trained data collectors from 1.12.2019–31.3.2020, anonymously with cooperation from nurses and physicians working in the medical centres. Data were obtained after approval by each institution and the Ethical Committee of the Faculty of Pharmacy, Comenius University Bratislava.

### Participants

Patients of either sex were included in this study based on the following inclusion criteria:

- age ≥ 65 years,
- at least 1 drug in treatment.
- date of the visit to the medical centre from 1.11.2019–31.12.2019, and.
- information about the presence of geriatric syndromes in patients’ records.

We excluded patients

- younger than 65 years, and/or.
- who did not use any medication, and/or.
- who did not come for a visit during the predefined period to the medical centre, and/or.
- had insufficient information about the presence of geriatric syndromes in their records.
- If a patient had more than 1 visit during the predefined observational period, the first occurrence was analysed.

### Variables and data sources

For statistical evaluation, basic demographic data (sex, age, attending physician), medical data (drugs used, daily dose) and comorbidities were collected.

The primary outcomes were the number of prescribed PIMs and polypharmacy. The number of drugs and PIMs used is expressed as the mean number for a patient per day (per day/patient). Secondary outcomes were the identification of the most frequently prescribed PIMs and number of PIMS vs. specialization of the attending physician.

All current prescriptions were recorded together with the doses. Drugs were identified as PIMs according to the EU(7) PIM list [21, 28], if they were long-term used (more than 3 months) at a determined dose. The insulin-sliding scale was not evaluated in this study due to a lack of information in medical records. PIMs were than classified according to the Anatomical Therapeutic Chemical (ATC) classification. Non-prescription (over-the-counter medications), dermal preparations, and medications on an as-needed basis were excluded.

Polypharmacy was defined as the concomitant use of at least five drugs [29, 30]. Excessive drug use was defined as the use of 10 or more concomitantly used drugs [31].

The risk of bias was assessed by the Cochrane Collaboration tool [32].

### Study size

The minimum sample size for our analysis was based on the estimated proportion. The sample size was set by using an online sample size calculator for a 95% confidence interval and a 5% margin of error [33]. The total number of elderly people aged 65 and over in 2019 in the Slovak Republic was 905 175 [34]; thus, the minimum number of included subjects was estimated to be 384. Our patient sample included 449 outpatients.

### Statistical methods

Variables were categorized as qualitative (sex, PIM use, polypharmacy, physician specialization (GP/INT/GER) or quantitative (age, number of prescribed drugs, number of prescribed PIMs). We set dichotomous primary dependent variable (at least one PIM versus no PIM). Independent variables (predictors) were sex (dichotomous variable) and attending physician. Covariate was the number of medications used.

Statistical analysis was performed using IBM SPSS v.19. The results are described by descriptive statistics and are expressed as frequencies (N), percentages [%] and arithmetical means with expression of standard errors of the means, minimum and maximum values and modes (MEAN ± S.E., [MIN-MAX; MODE]).

The relationships between the arithmetical means were evaluated using Student’s t test. Spearman’s correlation was used to describe the relationship between the number of drugs used and the number of PIMs. The strength of Spearman’s correlation was set as 0.00-0.19 to indicate very weak; 0.20–0.39 to indicate weak; 0.40–0.59 to indicate moderate; 0.60–0.79 to indicate strong and 0.80-1.0 to indicate very strong (p < 0.05) [35].

The relationship between qualitative variables (PIM use and polypharmacy) was evaluated using the chi-square, and p < 0.05 was considered statistically significant. To analyse the OR for PIM use in patients, we used binary logistic regression. The results are expressed as ORs with corresponding 95% confidence intervals (CIs).

For the analysis of significant predictors for PIM use, we created two nested (hierarchical) logistic regression models. Our first model was the association between PIM, attending physician and sex. The second model included covariates age and number of drugs used. For the overall fit of the models, Nagelkerke R Square and Hosmer and Lemeshow Test were used. The results of analysis are presented with regression coefficients (b), standard errors of the means (S.E.) and ORs with corresponding 95% CIs for both models.

In our analysis, no other multiple statistical method was used due to the limited number of covariates available for the analyses.

## Results

### General characteristics of the patients

Out of 449 patients included in our study, there were more women than men (68.2% vs. 31.8%, 306 vs. 143). The mean age of the patients in the study group was 76.1 ± 0.3 years [65–94; 68]. The mean age of women was comparable to that of men (W 76.6 ± 0.4 years vs. M 75.2 ± 0.6 years, p = 0.056).

The mean age of patients in the care of internists (74.1 ± 0.7 years) was significantly lower than that of patients in the care of GPs or GERs (GPs 76.7 ± 0.6 years, p = 0.012; GERs 76.7 ± 0.5 years, p = 0.006).

### Polypharmacy and PIM prescriptions

The mean number of all prescribed drugs in our study group was 6.7 ± 0.2 drugs per day/per patient [1–19; 4]. There was no difference between men and woman (M 6.7 ± 0.3 vs. W 6.8 ± 0.2 drug/day/patient; p = 0.794). We found no association between the number of prescribed drugs and patient age (ρ = 0.110, p < 0.05). Among all patients in our study, polypharmacy was present in 68.8% (309). The occurrence of polypharmacy was found to be similar among women and men (W 69% vs. M 68%). Excessive drug use (> 10) was found in 22.3% of patients (23% of women and 22% of men).

The mean number of drugs used for patients with polypharmacy was 8.4 ± 0.2 drugs/day compared to 3.1 ± 0.1 drugs/day/patient for patients without polypharmacy.

Among our patients, the total number of all identified PIMs was 748. The mean number of prescribed PIMs was 1.7 ± 0.1 PIMs per day/patient [0–12; 0]. A total of 73% of all patients used at least one PIM in their treatment regimen. There was no significant difference between women and men in the mean number of prescribed PIMs (W 1.7 ± 0.9 PIMs per day/woman vs. 1.7 ± 0.1 PIMs per day/man, p = 0.613). At least 1 PIM in therapy had 74% of men and 73% of women.

Among patients with PIMs in their treatment regimen, the mean number of PIMs used was 2.3 ± 0.1 [1–12; 1] PIMs per day/patient.

**Fig. 2:**
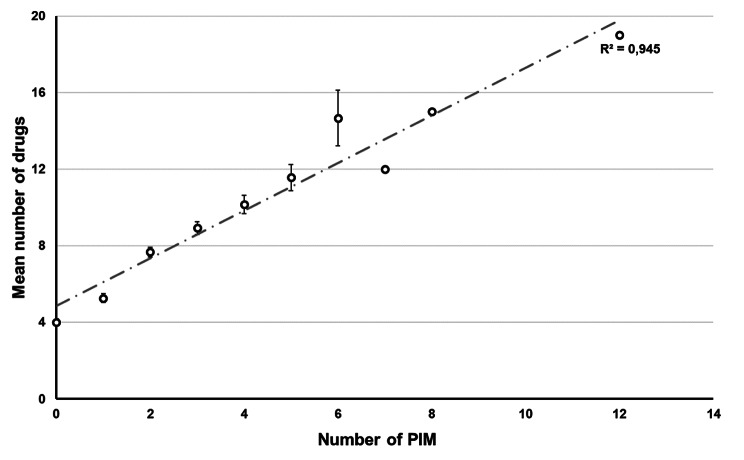
The relationship between the number of prescribed drugs and PIM.*PIM - potentially inappropriate medication*

We found a strong positive relationship between the number of drugs used and PIMs. The higher the number of prescribed drugs, the higher the incidence of PIM use (ρ = 0.690, p < 0.001, R^2^ = 0.945) (Fig. 2).

A significantly higher number of PIMs were included in the treatment regiments of patients with polypharmacy compared to patients without polypharmacy (2.2 ± 0.1 PIMs/day/patient vs. 0.6 ± 0.1 PIMs/day/patient, p < 0.001). We found that the odds of being prescribed PIMs were 6.7 times higher among those with polypharmacy than among those without polypharmacy (OR 6.7, 95% CI, 4.8–9.4, p < 0.001).

### The identification of the most frequently prescribed PIMs

Based on our results, pantoprazole was the most prescribed PIM among our patients. Table 1 lists the most often prescribed PIMs in our study.

**Table 1:** The list of the most prescribed drugs and PIM in patients in Slovak Republic

Drug	Patients using PIM (N = 449) (%)	Nb of PIM	Nb of PIM (N = 748) (%)
pantoprazole	14.9	67	9.0
urapidil	12.9	58	7.8
alprazolam	10.7	48	6.4
naftidrofuryl	9.1	41	5.5
ginkgo biloba	8.9	40	5.4
moxonidin	8.7	39	5.2
zolpidem	8.5	38	5.1
omeprazole	8.2	37	5.0
trimetazidine	6.0	27	3.6
apixaban	5.6	25	3.3
rilmenidin	5.6	25	3.3
verapamil	4.7	21	2.7
piracetam	4.5	20	2.6
bromazepam	4.2	19	2.5
theophylline	4.0	18	2.4

*Abbreviations: Nb – number, N – frequency, PIM - Potentially inappropriate medications*

**Fig. 3:**
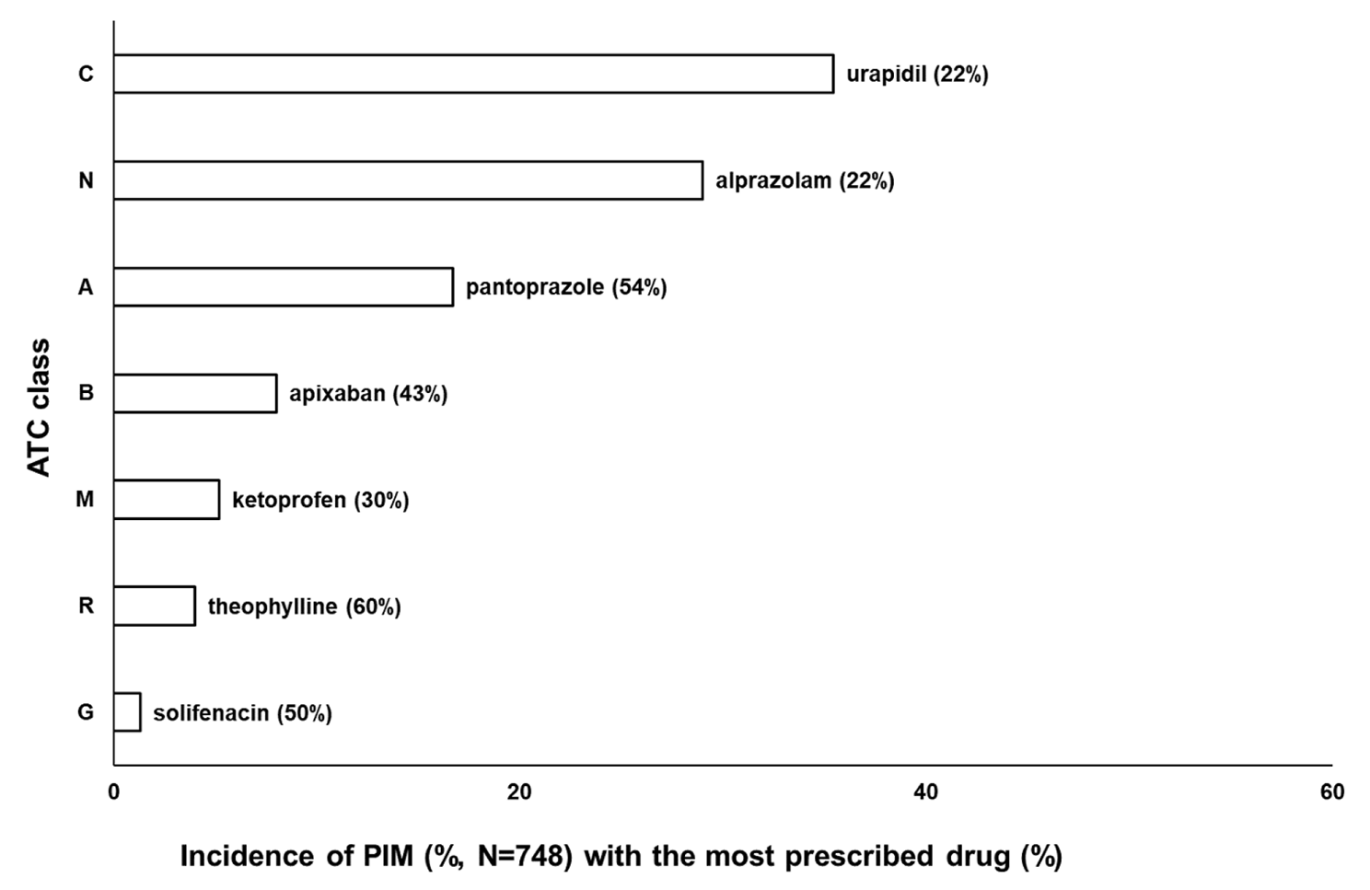
The most frequently prescribed PIM according to ATC classes and drugs in ambulatory outpatients. *PIM - potentially inappropriate medication*

Taking ATC classes into account, the most frequently prescribed ATC class was C (35.4% of PIMs), the drugs of which were used by 59.0% of patients who used PIMs, followed by classes N (29.0% of PIMs) and A (16.7% of PIMs) with 50.2% and 36.5% of patients, respectively. From ATC class C, the most frequently used PIM was urapidil, from ATC class N alprazolam and from ATC class A pantoprazole (Fig. 3).

The most frequent combination of 2 PIMs was alprazolam/pantoprazole and alprazolam/omeprazole. However, we also identified patient with 12 PIMs in therapy. This was an 83-year-old woman in the care of a GP. The PIMs she took were alprazolam, meloxicam, tramadol, theophylline, pinaverium bromide, ginkgo biloba, codeine, naftidrofuryl, omeprazole, solifenacin, diphenhydramine and clonazepam. In addition to these PIMs she took 4 more non-PIMs.

We found 6 PIMs uniquely present among our outpatients compared to our previous study in nursing homes [28]: aceclofenac, doxazosin, indomethacin, oxybutynin, pinaverium bromide and ranitidine.

### The specialization of the attending physician

Patients were in the care of a general practitioner (GP; 32.5%), internist (INT; 22.7%) or geriatrician (GER; 44.8%).

**Fig. 4:**
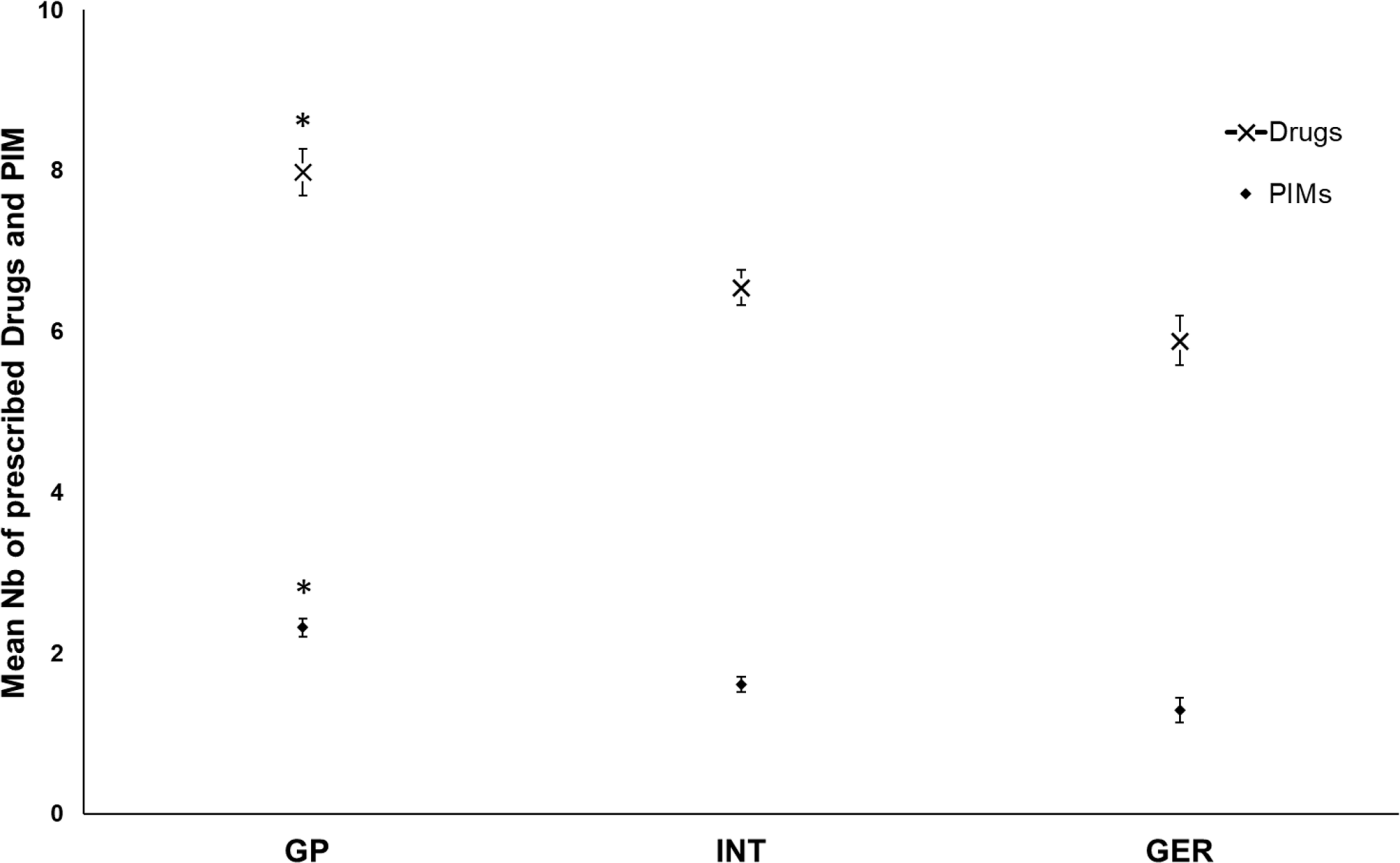
The prescription of drugs and PIM according to attending physicians. Abbreviations: GP ? general practitioner, INT ? internist, GER ? geriatrician, PIM ? potentially inappropriate medication, Nb ? number. The data are expressed as the mean ± standard error of the mean, *p<0.05 vs. INT and GER

We found a significantly higher number of drugs as well as PIMs prescribed by GPs than by INTs or GERs (Fig. 4). The mean number of prescribed drugs per day per patient was 8.0 ± 0.3 by GPs and 6.6 ± 0.3 INTs, while GERs prescribed only 5.9 ± 0.2 drugs/day/patient (GPs vs. INTs p = 0.003 and GERs p < 0.001). The mean number of prescribed PIMs per day per patient was 2.3 ± 0.2 by GPs, 1.5 ± 0.1 by INTs and 1.3 ± 0.1 by GERs (PIM/day/patient, p < 0.001 GPs vs. INTs and GERs). There were 4.2 times higher odds of being prescribed PIMs by GPs (OR 4.2, 95% CI, 2.4–7.3, p < 0.001) than by GERs, while the odds of being prescribed PIMs by INTs were only 2.0 times higher than the odds of being prescribed PIMs by GERs (OR 2.0, 95% CI, 1.2–3.5, p = 0.011). The differences in the prescription of PIMs regarding the specialisation of the attending physician are shown in Table 2. For a complete list of PIMd, see Additional File 1.

**Table 2:** The differences in the PIM use according to a specialisation of the attending physician

GPs	Incidence of PIMs (N = 338) (%)	INTs	Incidence of PIMs (N = 153) (%)	GERs	Incidence of PIMs (N = 257) (%)
alprazolam	8.6	urapidil	13.7	pantoprazole	11.3
zolpidem	6.8	pantoprazole	11.8	urapidil	9.7
naftidrofuryl	6.2	moxonidine	7.8	naftidrofuryl	6.2
omeprazole	6.2	ginkgo	6.5	trimetazidin	5.8
pantoprazole	5.9	verapamil	6.5	zolpidem	5.8
ginkgo	5.0	rilmenidine	4.6	alprazolam	5.4
moxonidine	4.7	omeprazole	3.9	ginkgo	5.1
piracetam	4.1	alprazolam	3.3	bromazepam	4.7
ketoprofen	3.6	apixaban	3.3	apixaban	4.3
urapidil	3.6	bromazepam	3.3	moxonidine	4.3

*Abbreviations: GP – general practitioner, INT – internist, GER – geriatrician, PIM – potentially inappropriate medication*

### Assessment of predictors for PIMs use

In our first model (the Likehood Ratio (LR) chi-square test LRχ^2^(3) = 29.6, p < 0.001) type of physician emerged as a positive and significant predictor (GPs b = 1.423, S.E.=0.3, OR 4.2, 95% CI (2.4–7.3), p < 0.001; INTs b = 0.697, S.E.=0.3, OR 2.0, 95% CI (1.2–3.5), p < 0.05 compared to GERs). Sex was not significant predictor in this model (b=-0.037, S.E.=0.3, OR 1.0, 95% CI (0.6–1.5), p = 0.876).

In the second model (the Likehood Ratio (LR) chi-square test LRχ^2^(5) = 144.9, p < 0.001) type of physician, age and number of drugs used emerged as the positive and significant predictors of prescription of PIMs (Table 3).

**Table 3:** The results for the second regression model with odds ratio for PIMs use

Covariates	b	S.E.	OR	95% CI	p-value
GPs*	1.135	0.33	3.1	1.6-6.0	0.001
INTs*	0.641	0.32	1.9	1.0-3.5	0.042
**Age**	0.049	0.02	1.1	1.0-1.1	0.009
**Number of drugs used**	0.493	0.06	1.6	1.5–1.8	0.000
**Sex**	-0.082	0.28	0.9	0.5–1.6	0.769

*Abbreviations: GP – general practitioner, INT – internist, PIM – potentially inappropriate medication, b - regression coefficient, S.E. - standard error, OR –odds ratio, CI – confidence interval*

* - compared to GERs

## Discussion

The EU(7) PIM list of potentially inappropriate medications represents the most comprehensive and up-to-date tool for assessing the prescribing of PIMs in use in Europe. In the present study, we focused on the evaluation of pharmacotherapy among geriatric outpatients with a focus on polypharmacy and the prescription of PIMs. Polypharmacy represents the strongest risk factor associated with PIM use [36]. This is usually because the multimorbidity of geriatric patients causes problems in controlling their diseases and preventing further complications [36]. Institutionalization or older age might be further determinants of the more frequent use of PIMs among the elderly individuals, as described in several studies [37, 38]. PIM use together with polypharmacy and multimorbidity may increase the risk of inappropriate prescriptions among elderly individuals [39].

In our study, out of 449 geriatric patients, polypharmacy was found in 68.8%. The mean number of drugs used was nearly 7 drugs per day. If we weight our data with those of other studies [40, 41], the rate of polypharmacy was high. However, in our previous study in nursing homes [28], we found a higher rate of polypharmacy (83%). Similarly, high polypharmacy rates were found in Italy [42] and France [43]. Furthermore, we found a positive relationship between the number of prescribed drugs and the number of prescribed PIMs. The higher the number of prescribed drugs, the higher the incidence of PIM use. Gallagher et al. found that patients taking > 5 medications were 3.3 times more likely to receive an inappropriate medication than those taking ≤ 5 drugs [8]. In our study, the odds of being prescribed PIMs were 6.7 times higher among those with polypharmacy (> 5 drugs used regularly) than among patients without polypharmacy. However, we found no association between the number of prescribed drugs and patient age. This could be due to the nonlinear relationship between age and the number of drugs taken, e.g., the mean number of drugs used among 73-year-old geriatric patients (n = 15) was 8 drugs per day/patient, while among 86-year-old geriatric patients (n = 10), it was 6.7 drugs per day/patient. Additionally, with age the number of patients decreased (e.g. there was only 1 patient who was 93 years old). We found that less than 5 drugs daily were used by 31% of patients. According to Jetha (2015), international research indicates substantially growing rates of polypharmacy and PIM use in the growing elderly population, and almost 50% take one or more medications that are not necessary. Furthermore, the International Group for Reducing Inappropriate Medication Use and Polypharmacy published a position statement with 10 recommendations for action to reduce inappropriate medication use and polypharmacy [44]. Fried and Mecca (2019) presented a more complex view on polypharmacy among elderly individuals. They described the concept for appropriate polypharmacy by stressing the problem of underutilized versus inappropriate medicine use among elderly individuals [45].

The prevalence of PIM use depends on the criteria used, national prescription habits, and the studied population. Our previous study showed that more than half (53.2%) of 282 potentially inappropriate medications listed in the EU(7) PIM list are authorized in the Slovak Republic [28]. This number is comparable with those in other European countries, such as Hungary (54.4%), the Czech Republic (49.1%) and Serbia (42.4%). However, this number is lower that in Spain (70.7%) or Portugal (66.4%) [46]. Out of all available PIMs in the SR, we identified 69 (24.5%) used among outpatients in this study, stressing certain prescription habits.

At least one PIM according to the EU(7) PIM list was found in 77.3% of residents of care homes in France [43], 40.9% in Swedish hospitals [47], 54.2% of community-dwelling patients in Albania [48], and 37.4% in Germany [49]. In our study, 73% of outpatients used at least one PIM, which is less than we found in our previous study in nursing homes (almost 91%) [28]. We found no differences between men and women in the number of PIM used. Nevertheless, these numbers are rather high and might be due to the lack of national guidelines for appropriate prescribing for elderly individuals.

The drugs from ATC classes C, N and A were together the most prescribed PIMs and at the same time the most prescribed ATC classes in Slovakia in 2019 [50]. The spectrum of PIMs used for outpatients in this study was similar to that used for nursing home patients [28]. The main differences were in ATC class C and ATC class N. Among nursing home patients [28], the most prescribed PIMs in ATC class C were naftidrofuryl (13.6%), digoxin (8.4%) and trimetazidine (5.5%); among outpatients in this study, these were urapidil (12.9%), naftidrofuryl (9.1%) and moxonidine (8.7%). The characteristics of our data did not allow us to consider individual patient characteristics, although the two most represented diagnoses were essential hypertension (46%) and chronic ischaemic heart disease (33%). However, of note was the frequent use of naftidrofuryl. The effect of this medicine is rather symptomatic, and the long-term effect on minimum walking distance among patients with claudication/peripheral vascular disease is not known [51]. Antidementia drugs are a safer alternative for elderly individuals who might benefit from vasodilation in the cranial region [18].

Regarding ATC class N, we found that 23 different PIMs were used among patients. According to Abraham et al., 30% of PIMs may exacerbate cognitive impairment in elderly individuals [52]. Overuse of antipsychotics is associated with an increased risk of hospitalizations, cardiovascular events, hip fractures and death [53], although antipsychotics are used in schizophrenic and bipolar patients. In our study, we identified use of 5 antipsychotic PIMs (quetiapine, haloperidol, risperidone, olanzapine and tiapride) among 26 patients (5.8%). We found proper indication for their use only among 4 patients. However, except for haloperidol (GPs) and tiapride (INTs and GERs) antipsychotics are prescribed only by psychiatrists, we assume that patients were in care of psychiatrists as well, only the information about their diagnosis were missing in records. However, there is a great need for nonpharmacological interventions for mental health problems in geriatric patients [54].

Setting up the appropriate treatment regimen for elderly people requires not only theoretical knowledge but also clinical judgement and experience [40]. Geriatricians are more aware of potentially inappropriate medications and may identify, replace or deprescribe PIMs more frequently than internists or general practitioners [37]. GPs are usually responsible for long-term follow-up and repeat prescriptions [55], while geriatricians are skilled in the treatment of physiological and pathological changes connected with ageing [56]. In Slovakia, the competencies of physicians are limited by prescription restrictions, and GPs have the most limited prescription options. On the one hand, they have the most comprehensive view of all prescriptions of the individual patient and they can contact specialists and discuss the appropriateness of prescribed PIMs with them. Of the 10 most prescribed PIMs in our study, GPs cannot prescribe urapidil (the 10th most prescribed PIM, Table 2). Despite this, we found in our study significantly higher number of prescribed drugs as well as PIMs by GPs than by GERs. The mean number of prescribed drugs per day per patient was almost 8 by GPs, while by GERs, it was nearly 6 (p < 0.05). Similarly, the mean number of prescribed PIMs by GPs was 2.3, while that by GERs vas 1.3 (p < 0.05). We found 4.2 times higher odds of being prescribed PIMs by GPs than by GERs (p < 0.001). Considering patient sex, age, and number of drugs used; GPs, INTs and number of drugs used had greater odds for PIM prescription. Sex was not significant predictor. However, adjusting the data to other patient parameters e.g., polymorbidity, BMI or other factors like prescription habits of physicians or their age might alter these findings as well.

According to Gnjidic et al., the use of five medications or more is associated with frailty and disability. Frailty and disability might be the most relevant grounds to seek care in nursing homes. The increased severity of conditions in elderly people reduces the variability in treatment options and leads to specific prescription habits [44]. In 2019, there were 128 geriatricians in the Slovak Republic [57], while the number of individuals aged 65 years and older was 905 175 [34]. Since the care for a geriatric patient is usually in the hands of a GP or internist, in many cases the intervention of a geriatrician is unavailable. As we showed in this study, the role of geriatricians in the proper treatment of geriatric patients is essential. The Slovak Republic lacks GPs, and their gatekeeping role is weak. That might be an opportunity for pharmacists, as they rank among the most approachable and accessible health care providers in Slovakia. After special training on safe pharmacotherapy in vulnerable elderly individuals, pharmacist might be an appropriate partner for communication with GPs about pharmacotherapy in geriatric patients. Thus, trained pharmacists or clinical pharmacists might be valuable members of the multidisciplinary team in elderly care [40, 58, 59].

### Limitations of this study

The definition of PIMs indicates that drugs listed in PIM lists are not contraindicated but might be inappropriate for an individual patient. However, in some cases, their use is justified and might be acceptable.

The use of explicit criteria, such as the EU(7) PIM list, is limited by their single drug/disease-oriented approach, since both explicit and implicit approaches might be preferred if possible.

In our study, we assessed pharmacotherapy retrospectively and comprehensively; however, we did not assess patients individually based on their clinical condition. Due to the limited availability of clinical and patient history data, multifactorial analysis could not be performed.

The simple design of our study allowed us to adhere to our main aims; on the other hand, it limited the number of outcomes. There was no space for education or interventions about PIM use among physicians.

Polypharmacy is associated with PIMs, which was shown in our data. However, arbitrary use of the total number of medications as a diagnostic test for the quality of care is dangerous. More complex models are needed.

## Conclusion

Due to population ageing, safe pharmacotherapy for elderly individuals has become a societal priority. With the use of explicit criteria, our study briefly described the situation and identified the main problems as the high rates of polypharmacy and most frequently used PIMs from ATC classes C, N and A. Even though most of the patients were in the care of geriatricians, we identified the highest odds for being prescribed PIMs by GPs. However, the use of multifactorial analysis might alter these results.

Similar to other European countries, population ageing in Slovakia creates opportunities for health policy-makers as well as universities to set up a safe system of health care for elderly people involving systematic methods such as medication reviews in multidisciplinary teams that include pharmacists and clinical pharmacists.

### Electronic supplementary material

Below is the link to the electronic supplementary material.

Supplementary Material 1

## Data Availability

Data are included in Additional file 1 in the manuscript. Other data that support the findings of this study are available upon reasonable request from the corresponding author [T.F.].

## Abbreviations

PIM: potentially inappropriate medication
OUT: geriatric outpatients
GP: general practitioner
GER: geriatrician
INT: internist
ATC: Anatomical Therapeutic Chemical Classification System
W: women
M: men
EU(7) PIM list: European list of PIM ? EU(7)
Nb: number
b: regression coefficient
S.E.: standard error
OR: Odds ratio
CI: confidence interval
